# Case Report: Local Cytokine Release Syndrome in an Acute Lymphoblastic Leukemia Patient After Treatment With Chimeric Antigen Receptor T-Cell Therapy: A Possible Model, Literature Review and Perspective

**DOI:** 10.3389/fimmu.2021.707191

**Published:** 2021-07-19

**Authors:** Chengxin Luan, Junjie Zhou, Haixia Wang, Xiaoyu Ma, Zhangbiao Long, Xin Cheng, Xiaowen Chen, Zhenqi Huang, Dagan Zhang, Ruixiang Xia, Jian Ge

**Affiliations:** ^1^ Department of Hematology, The First Affiliated Hospital of Anhui Medical University, Hefei, China; ^2^ Institute of Translational Medicine, The Affiliated Drum Tower Hospital of Nanjing University Medical School, Nanjing, China

**Keywords:** local cytokine-release syndrome, acute lymphoblastic leukemia, possible model, systemic cytokine-release syndrome, chimeric antigen receptor T therapy

## Abstract

Chimeric antigen receptor T (CAR-T) cell therapy has achieved remarkable clinical efficacy in treatment of many malignancies especially for B-cell hematologic malignancies. However, the application of CAR-T cells is hampered by potentially adverse events, of which cytokine release syndrome (CRS) is one of the severest and the most studied. Local cytokine-release syndrome (L-CRS) at particular parts of the body has been reported once in a while in B-cell lymphoma or other compartmental tumors. The underlying mechanism of L-CRS is not well understood and the existing reports attempting to illustrate it only involve compartmental tumors, some of which even indicated L-CRS only happens in compartmental tumors. Acute lymphoblastic leukemia (ALL) is systemic and our center treated a B-cell ALL patient who exhibited life threatening dyspnea, L-CRS was under suspicion and the patient was successfully rescued with treatment algorithm of CRS. The case is the firstly reported L-CRS related to systemic malignancies and we tentatively propose a model to illustrate the occurrence and development of L-CRS of systemic malignancies inspired by the case and literature, with emphasis on the new recognition of L-CRS.

## Introduction

Chimeric antigen receptor T (CAR-T) cells, which are genetically modified T cells harvested from patients to express special CARs, target tumor cells and can produce remissions again for patients who are resistant to standard therapies (1). CAR-T cell therapy has demonstrated inspiring efficacy in the treatment of B-cell original malignancies and its scope of application is broadening to many other malignancies even to solid cancers (2–6). Nevertheless, despite its promising efficacy, adverse events such as cytokine-release syndrome (CRS), immune effector cell-associated neurotoxicity syndrome (ICANS), cytopenia and B-cell aplasia have limited the application of CAR-T cell therapy (7). Being one of the most common and severest adverse event, CRS is regarded as a systemic inflammatory reaction with constitutional manifestation such as fever and hyoxemia (8). Recently, some reported that CRS can take place in particular regions of the body after treatment with CAR-T cell therapy for malignancies such as ovarian cancer and B-cell lymphoma (9, 10). Local CRS (L-CRS) is suggested as its medical term and Wei et al. have proposed a model illustrating the occurrence and evolution of CRS related to CAR-T cell therapy in B-cell non-Hodgkin lymphoma (B-NHL) (11). The model attempts to show the basic biology of CRS in B-NHL and indicates that L-CRS is a pre-stage of systemic CRS (S-CRS), which facilitates the understanding and management of CRS. However, the existing literature reported only about compartmental tumors, and some speculated L-CRS cannot happen in systemic malignancies (11). Our center took in a 61-year-female with relapsed B-cell acute lymphoblastic leukemia (B-ALL), a systemic malignancy ideal for CAR-T treatment, and she achieved a complete remission (CR) by CAR-T 19 cell therapy (A clinical trial in our center, registration number: ChiCTR1800016315, cells provided by the Gracell Biotechnologies of Shanghai). After CAR-T infusion, the patient exhibited life threatening dyspnea by the rapid enlargement of her neck which was successfully mitigated with tocilizumab and corticosteroids. L-CRS was under suspicion and the case is the firstly reported L-CRS related to systemic malignancies and we tentatively propose a possible model to illustrate the occurrence and development of L-CRS of systemic malignancies inspired by the case and literature, with emphasis on the new recognition of L-CRS, and caution drawn to systemic malignancies for possible L-CRS.

## Case Presentation

**Figure 1:**
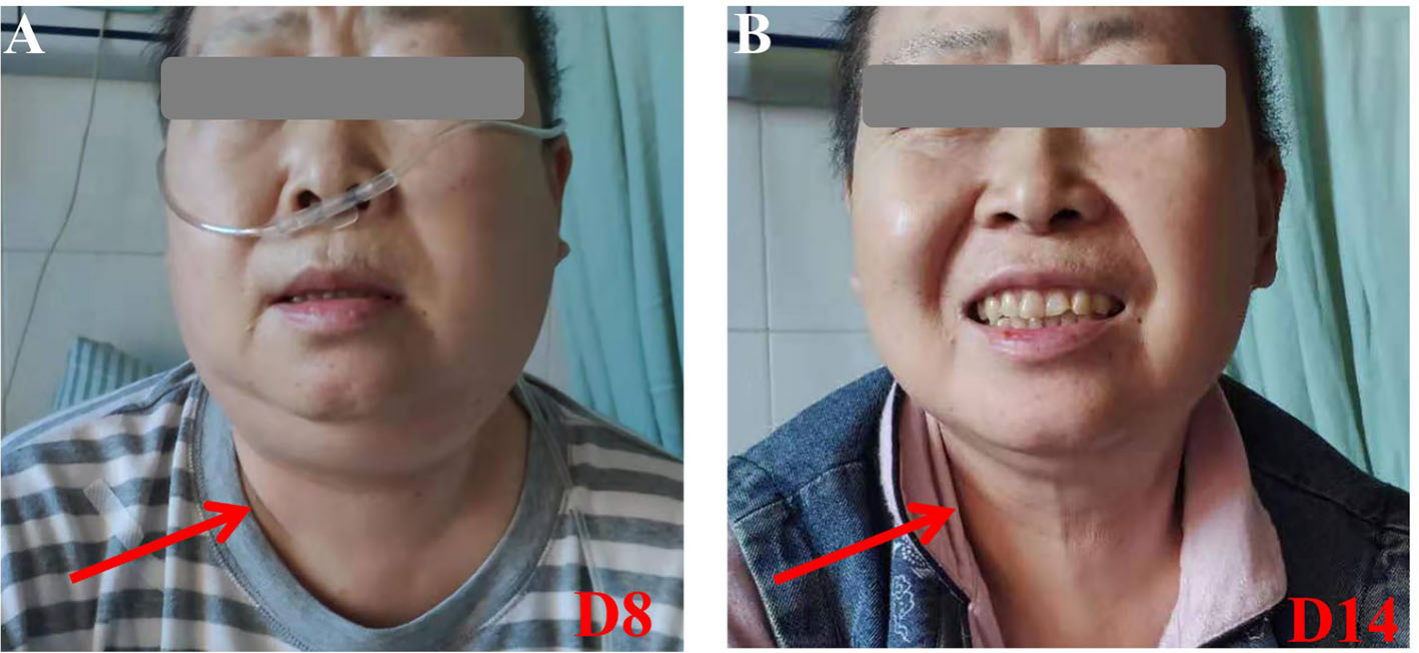
Pictures of the cervical region. **(A)** At D8, her cervical region was obviously swollen. **(B)** At D14, her cervical region returned to normal. With consent to publish from the patient.

**Figure 2:**
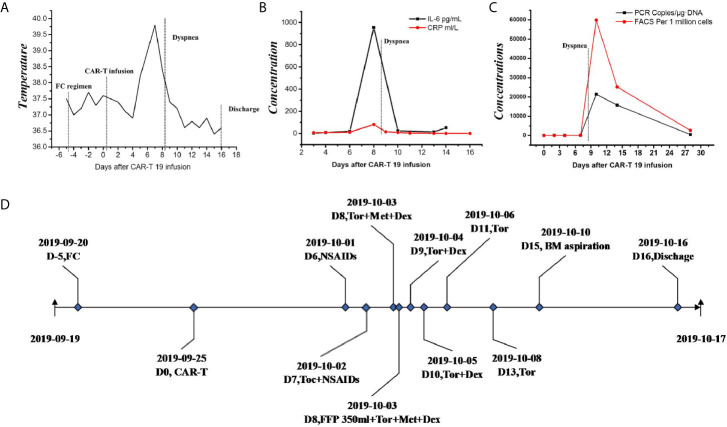
Clinical data of L-CRS. **(A)** The temperature profile. **(B)** levels of cytokine. **(C)** CAR-T copy number. **(D)** therapeutic process. FC, fludarabine and cyclophosphamide regimen; IL-6, interleukin-6; CRP, c reactive protein; PCR, polymerase chain reaction; FACS, fluorescence activated cell sorter; NSAIDs, non-steroidal anti-inflammatory drugs; Toc, tocilizumab; Tor, torasemide; Met, methylprednisolone; Dex, dexamethasone; FFP, fresh frozen plasma; BM, bone marrow.

A 61-year-female of Han nationality was admitted to our center for muscular soreness and motor disorder of both legs. Physical examination (PE) indicated an anemic face, but no obvious infiltration manifestation such as swollen gums, enlarged lymph glands, sternal tenderness or hepatosplenomegaly. Blood test revealed that the count of white blood cell (WBC) was 348.43 × 10^9^/L, incapable of differential count, level of hemoglobin (HGB) was 44 g/L and the number of platelet (PLT) was 35 × 10^9^/L. Hematologic malignancies were suspected and bone marrow aspiration was performed. She was diagnosed with Ph-like B-ALL (Poor risk, Philadelphia chromosome-like with IKZF and SH2B3 mutation, common-B with CD33 expression). She accepted pretreatment with dexamethasone, inducing chemotherapy with imatinib + VDP (vindesine, daunorubicin and dexamethasone) regimen and she achieved a CR. Then she was monitored by flow cytometry (FCM) as minimal/measurable residual disease (MRD) surveillance and consolidated with imatinib + VDP, imatinib + MTX (methotrexate) + pegaspargase, imatinib + hyper-CVAD (hyper fractionated cyclophosphamide, vincristine, doxorubicin, and dexamethasone) as well as central nervous system (CNS) prophylaxis (methotrexate, cytarabine and dexamethasone). In June 2019 bone marrow aspiration showed that the proportion of lymphoblast was 34%, blood test revealed that the count of WBC was 17.03 × 10^9^/L, HGB 102 g/L and PLT 197 × 10^9^/L, serum biochemical indicators revealed lactate dehydrogenase (LDH) was 399 U/L, the disease was confirmed to be relapsed and she was re-induced with imatinib + VILP (vindesine, idarubicin, pegaspargase and dexamethasone), to which the patient did not respond (MRD:26%). She is a high-risk ALL and clinical trials were recommended. The patient and her relatives understood her condition and comprehended that clinical trial or hematopoietic stem cell transplantation (HSCT) was her only chance. Later the patient was enrolled in a CAR-T 19 clinical trial in our center (Registration number: ChiCTR1800016315, provided by the Gracell Biotechnologies of Shanghai) and a sequential regimen of dasatinib + VIP (vindesine, idarubicin and dexamethasone) was applied before CAR-T, after which she still in non-remission (MRD: 30%). From the first diagnosis to the time before CAR-T, the patient had no complain of pain or mass of the whole body, and all CT scan for head, chest, mediastinum, abdomen, pelvis and B-ultrasonography for abdomen showed no evidence of cervical invasiveness or extramedullary infiltration. PE before CAR-T revealed no abnormality such as swollen gums, enlarged lymph glands, sternal tenderness and hepatosplenomegaly. Therefore, extra medullary disease (EM) was not considered. Blood test before CAR-T pretreatment revealed that the count of WBC was 2.26 × 10^9^/L, lymphocytes 66.81%, HGB) 57 g/L and PLT 280 × 10^9^/L, serum biochemical indicators revealed LDH was 314 U/L. Under informed consent, she was given pretreatment with FC (fludarabine and cyclophosphamide) regimen on 2019.9.20 to clear lymphocytes and a dose of 1.165 × 10^5^ CAR-T cells per kilogram modified to anti-CD19 was infused on 2019.9.25 (d0, autologous CAR-T cells with costimulation molecule CD28). Within 12 h of CAR-T19 infusion, the patient developed a low-grade fever to 37.5°C, which rose to 39.0°C with blood pressure drop about 20 mmHg and inflammatory cytokines surging at d6 after the CAR-T infusion, grade 2 CRS was considered [the American Society for Transplantation and Cellular Therapy (ASTCT)] (12). She got fluid boluses and oxygen inhalation treatment (low-flow nasal cannula). Non-steroidal anti-inflammatory drugs (NSAIDs) were instituted as symptomatic treatment and cefmetazole was used in case of infection at d6. At d7, as her temperature rose to 39.8°C, tocilizumab (8 mg/kg) was administered and anti-infection regimen was upgraded to imipenem for possible life threating infection. Her temperature dropped to about 38.0°C and blood pressure returned to baseline from d7. Her condition seemed to be improving, however, at 17:10 of the D8, she complained of foreign body sensation in the larynx, dyspnea and facial edema with neck circumference growing rapidly (shown by **Figure 1A**). Blood test revealed that the count of eosnophils was 0.07 × 10^9^/L (1.44%), basophil 0.00 × 10^9^/L (0.04%), which indicated no allergic reaction. Creatinine was 38.4μmol/L, glomerular filtration rate (GFR) was 115 ml/min, alanine transaminase (ALT) was 33 μ/L, brain natriuretic peptide (BNP) was 43.4 pg/ml, which indicate normal function of kidney, liver and heart. Urgent measures were taken, she received oxygen inhalation (high-flow nasal cannula), torasemide 5 mg, methylprednisolone 40 mg and dexamethasone 10 mg continuously, with which relieve symptoms other than the swollen neck were achieved. About 5 h later, torasemide 5 mg, methylprednisolone 40 mg and dexamethasone 10 mg were repeated and fresh frozen plasma 350 ml was infused. The neck circumference was decreasing and the dosage of corticosteroids was reduced accordingly. At d11, the swollen neck almost subsided and corticosteroids were stopped (**Figure 1B**). Bone marrow aspiration at d15 revealed a CR, upon which the patient was discharged at d16. The temperature profile, CRS levels of cytokine, CAR-T copy number and therapeutic process of CRS are presented by **Figures 2A–D** respectively. Then follow-up was arranged for her and MRD was monitored. She remained in CR about one year and refused to conduct HSCT, which she considered risky and expensive. In August 2020 bone marrow aspiration showed that the proportion of lymphoblast was 11%, she had relapsed again and received two cycles of dasatinib + VIP regimens, to which she did not respond and died of respiratory failure in November 2020.

### Discussion, Model Proposal, and Perspective

**Figure 3:**
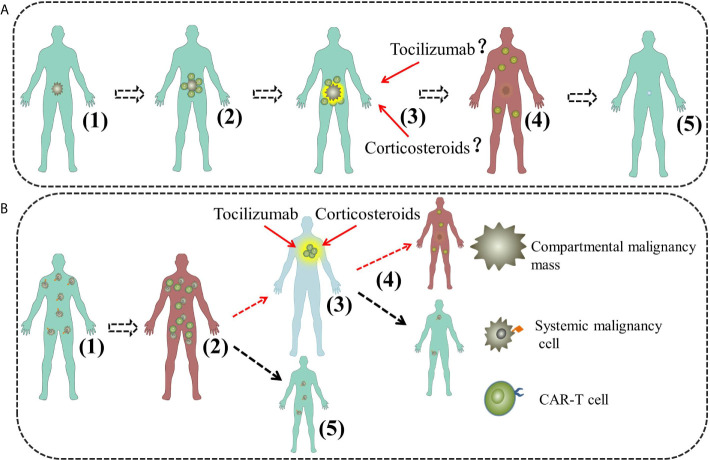
The model of L-CRS for compartmental and systemic malignancies. **(A)** The model of L-CRS for compartmental malignancies. (1) Malignancy cells restricted; (2) First stage, CAR-T cell local expansion; (3) Second stage, CAR-T cell overflow and inflammatory cytokine surge; (4) Third stage, CAR-T cell redistribution and organ damage; (5) Fourth stage, recovery. **(B)** The model of L-CRS for systemic malignancies in our case. (1) Malignancy cells diffused. (2) First stage, S-CRS; (3) Second stage, CAR-T cell redistribution and organ damage, possible for L-CRS; (4) Third stage, CRS abatement and recovery, possible for a new cycle of S-CRS; (5) In most case, L-CRS does not happen or just too weak to be identified.

CRS is driven by cytokines released by the infused CAR-T cells and amplified by activated monocytes and macrophages. The cytokines are of variety and interleukin-6 (IL-6) is regarded as the core factor, others such as interferon-γ (IFN-γ), interleukin-2 (IL-2) and interleukin-2 (IL-10) as contributors (13, 14). However, the clear mechanism of CRS secondary to CAR-T cell therapy is elusive and no model manages to thoroughly illustrate its clinical manifestations (15). Though emerging evidences have proven that IL-6 is the central mediator of CRS secondary to CAR-T cell therapy or other immunotherapy, IL-6 targeting therapeutic strategy such as tocilizumab fails in some cases, which indicates CRS is heterogeneous and more researches are needed to elucidate its underling mechanism (11). For this purpose, clinical observation, summary and speculation give important clues. CRS used to be regarded as a systemic syndrome until a few reports indicated that CRS could be confined to certain regions of body. Tanyi et al. described a compartmental CRS in a patient with ovarian cancer following autologous mesothelin targeted CAR-T cell therapy (9). Jin et al. reported a successful treatment of a diffuse large B-cell lymphoma (DLBCL) patient suffering from severe dyspnea during CAR-T19 cell therapy (10). Recently, Wei et al. summarized their practical clinical experience and reviewed the existing clues, then they proposed a model to illustrate the occurrence and progression of CRS for B-NHL, L-CRS was brought up as the medical term for the new type of CRS in accordance with the widely recognized S-CRS (11). As shown in **Figure 3A**, the model divided CRS into four stages: First stage, CAR-T cell local expansion stage [**Figure 3A-(2)**]; Second stage, CAR-T cell overflow and inflammatory cytokine surge stage [**Figure 3A-(3)**]; Third stage, CAR-T cell redistribution and organ damage stage [**Figure 3A-(4)**]. Fourth stage, recovery stage [**Figure 3A-(5)**]. L-CRS is regarded as the early stage of CRS for B-NHL due to the aggregation and local expansion of the infused CAR-T cells in the tumor masses, where there will be a large number of cytokines release when CAR-T cells catch and kill tumor cells. Local inflammatory response driven by CAR-T cells or contributed by other immune cells occurs and L-CRS ensues. After the wiping-out of tumor cells, locally expanded CAR-T cells and cytokines shift to circulatory system due to the loss of primary target locally, which marks the second stage with S-CRS developed. Without antigen stimulation, the proliferation of CAR-T cells is retarded and CRS enters the third stage and possibly the fourth stage. Obviously, the model is based on the premise that the malignancies must be confined to a certain region of the body and L-CRS happens within. Besides, supported by the existing reports, the study also suggested that L-CRS can only take place in compartmental malignancies. The model is helpful for understanding and managing L-CRS, however, L-CRS is rarely studied and caution should be paid to non-compartmental malignancies.

In this paper, we presented a successful treatment of an ALL patient suffering from severe dyspnea during the course of CAR-T cell therapy. The patient had no previous allergic reactions history to food or any drugs and the blood test at D0–D9 revealed no evidence of allergic reaction (without eosinophils or basophils increase). Her cardiac function, hepatic function and renal function had no obvious abnormity. L-CRS was a possible and reasonable explanation for her clinical manifestation. To our knowledge, this is the first report of L-CRS in systemic malignancies. Interestingly, L-CRS happened during the course of grade-2 S-CRS in our case, which suggests a different mechanism for this intriguing phenomenon. Inspired by our case and literature, we propose another possible model to illustrate the underling mechanism of L-CRS in systemic malignancies.

As shown by **Figure 3B**, our model comprises three stages, First stage, S-CRS stage [**Figure 3B-(2)**]: CAR-T cells expand, effect and release inflammatory cytokines; Second stage, CAR-T cell redistribution and organ damage stage, L-CRS may happen [**Figure 3B-(3)**]; Third stage, CRS abatement and recovery stage [**Figure 3B-(4)**]. After infusion, CAR-T cells are activated and expanding after interaction with their target cells which are distributed all over the body, then they kill the tumor cells and release a large number of cytokines, consequently, systemic inflammatory response is triggered and S-CRS develops. Having eliminated the tumor cells in most sites of body, CAR-T cells move on to the sites that could harbor tumor cells or just catch other cells owing to the “off-target effect’’ as tissues may express antigens same to that on tumor cells hence can also be recognized (16). In our case, L-CRS happened in cervical region where lymphoid tissues prevail and could harbor the tumor antigens or the tumor cells. S-CRS was relatively milder compared with the ensuing L-CRS in the case, which indicates L-CRS can be more severe and calls for more attention and preparation of effective measures. Though in most cases L-CRS either does not happen or presents too mildly to be identified [**Figure 3B-(5)**], severe L-CRS may as well contribute to a new cycle of S-CRS if it does not just abate [**Figure 3B-(4)**].

Due to unique features of L-CRS in systemic malignancies followed by CAR-T therapy, it stands to reason that the management strategy should be different from that for the compartmental ones. As mentioned above, IL-6 is considered to be the central factor for the development of CRS. In most cases, blockade of receptor of IL-6 (IL-6R) is effective and recommended by guides for controlling CRS of immune therapy as well as CAR-T. Tocilizumab is the most used blocker of IL-6R, but researchers have reported about cases unresponsive to it (11, 17). The phenomenon can be explained at molecular level by the reported researches. IL-6 has two sides, which depends on the receptors that it combines with. There are two types of IL-6R, membrane form of interleukin-6 receptor (mIL-R) and soluble form of interleukin-6 receptor (sIL-R). mIL-R is restricted to IL-6R-expressing cells including macrophages, neutrophils, T cells, and hepatocytes. When level of IL-6 is low, IL-6 predominately binds to mIL-R and leads to anti-inflammatory activities, namely classic IL-6 signaling. When the level of IL-6 is elevated, IL-6 predominately binds to sIL-R; the IL-6/sIL-R complex then binds to gp130 (membrane glycoprotein 130), a protein that is ubiquitously expressed on cytomembrane and leads to pro-inflammatory activities, namely trans-IL-6 signaling (13, 18). On this account, the blockade of IL-6R at early stages of CRS may be counterproductive, which have been noted by some clinical observations (11). For L-CRS of compartmental malignancies such as B-NHL, this mechanism clarifies that L-CRS could be aggravated by the use of tocilizumab. While for L-CRS of systemic malignancies such as ALL, exampled by our case, L-CRS is at advanced stage, therefore, tocilizumab can be used.

Corticosteroids have been shown to significantly suppress T-cell function and induce T-cell apoptosis, therefore, most treatment algorithms consider corticosteroids as second-line therapy for CRS because of concerns that the use of corticosteroid can lead to impairment of therapeutic effect of CAR-T cells. For example, the revised CRS grading system by Lee et al. only recommend the use of corticosteroids for CRS higher than grade 2 and irresponsive to tocilizumab (12, 13).

While recently some researches indicated earlier steroid use could not affect efficacy of CAR-T, and may reduce the rates of CRS (19, 20). Despite the controversy, in compartmental malignancies such as B-NHL, corticosteroids may be used cautiously for L-CRS, which develops at an early stage when corticosteroids could produce significant inhibition of the CAR-T therapy unless life threating. While for systemic malignancies such as our case, L-CRS happens at advanced stage and the reduction of therapeutic effect can be bearable if not totally negligible, therefore, corticosteroids even ictus treatment of methylprednisolone should be considered.

In conclusion, we tentatively proposed a treatment algorithm for L-CRS of compartmental and systemic malignancies shown by **Figure 3**. Of note, L-CRS caused by CAR-T cells are diverse and not fully understood, factors like malignancy type, location, tumor burden, doses or type of CAR-T and so on may be related to the occurrence and severity of L-CRS. Moreover, some information such as histological proof, positive PET-CT scan of the cervical region at time of the swelling is missing due to the emergency of the case. Therefore, the model proposed by us may be arbitrary and inconclusive, more researches are needed to fully understand the underlying mechanism of L-CRS. With increasing use of CAR-T cell therapy, it is necessary to understand its side effects especially for the unusual one. This paper reminds us that patients with systemic malignancies such as ALL can also suffer from L-CRS and they should be carefully evaluated for possible emergency care needed for proper countermeasures such as vigilant monitoring, aggressive supportive or immunosuppressive agents.

## Data Availability Statement

The original contributions presented in the study are included in the article/supplementary material. Further inquiries can be directed to the corresponding author.

## Ethics Statement

Written informed consent was obtained from the patient and her legal guardian for the publication of any potentially identifiable images or data included in this article.

## Author Contributions

CL was one of the main health care providers of the patient,collected, analyzed the data, and wrote the paper. JZ was one of the main health care providers of the patient and collected the data. HW, XM, ZL, XC, XWC, and ZH were health care providers of the patient. DZ helped revise the paper. JG and RX were in charge of the related clinical trial and revised the paper. All authors contributed to the article and approved the submitted version.

## Funding

This work was supported by the Natural Science Foundation of Anhui province (Grant No.2008085QH365); Key Research and Development Project of Anhui Province (Grant No. 201904a07020057); Research Foundation of Anhui Medical University (Grant No. 2020xkj166).

## Conflict of Interest

The authors declare that the research was conducted in the absence of any commercial or financial relationships that could be construed as a potential conflict of interest.
